# “Blood lead level among battery factory workers in low and middle-income countries: Systematic review and meta-analysis”

**DOI:** 10.3389/fpubh.2022.970660

**Published:** 2022-10-05

**Authors:** Ararso Tafese Olana, Abera Kumie, Teferi Abegaz

**Affiliations:** Department of Preventive Medicine, School of Public Health, College of Health Sciences, Addis Ababa University, Addis Ababa, Ethiopia

**Keywords:** battery factories, blood lead level, low and middle-income countries, work, lead

## Abstract

**Introduction:**

Lead is one of the most health-hazardous causes of acute and chronic poisoning at workplaces. A limited study was conducted on the blood lead concentration among battery factory workers in low and middle-income countries. Therefore, this study will improve workplace health and safety conditions of workers in this sector and serve as baseline data for further studies in this segment of the working setup.

**Objective:**

This review aims to identify the pooled mean blood lead level among battery factory workers in low and middle-income countries.

**Methods:**

The search methods considered the following electronic bibliographic databases: google scholar, PubMed, and other gray literature. A funnel plot and Begg test were used to see the publication bias. The heterogeneity of studies was checked using I-square statistics with a cut of point 75% and the Joanna Briggs Institute (JBI) quality assessment tool was applied to ensure the quality of the included articles. A random-effect model was applied to pool the blood lead level intoxication. The sub-group analysis and Meta-regression analysis were conducted by country and year of publication to control heterogeneity and to show variation. We included the articles published from 2000 to 2021 year in the English language.

**Results:**

Through the search strategies, 135 articles were identified and 43 full-text articles were selected for evaluation, and finally, eighteen (18) articles fit the inclusion criteria. From the 18 studies included in the meta-analysis, the mean pooled blood lead level of workers was 37.996 μg/dl (95% CI: 30.680–45.312) which is higher than the threshold limit value set by American conference of governmental industrial hygienists (20 μg/dl). In subgroup analysis by year in the random effect model, the pooled mean of blood lead level from 2006 to 2011= 43.20 μg/dL (35.91–50.50), 2012–2015 = 37.78 μg/dl (25.23–50.29), and 2016–2020 = 36.53 μg/dl (19.44–53.62).

**Conclusions:**

This review showed that the pooled mean blood lead level of workers exposed to lead battery factories was (37.996 μg/dl) which is above the threshold limit value (20 μg/dl). Therefore, attention should be given by employers, government, and researchers to improve the health of working populations exposed to lead exposure in low and middle-income countries through the provision of occupational health and safety services like periodical medical checkups, treatments, and provision of training and adequate and appropriate personal protective equipment.

**Systematic review registration:** Identifier: CRD42022322827.

## Introduction

Lead is one of the common oldest health hazards known more than 2000 years ago which causes acute and chronic poisoning at workplaces (1, 2). It is considered a health issue for the general population through the pollution of the environment (3). It is heavy poisoning metal that causes harmful effects when it enters the human body by either ingestion, inhalation, or dermal contact (4). Lead poisoning is a serious threat to human health, specifically to employees exposed to lead in their daily activities. It is one of the causes of occupational disease in which workers developed serious complications in some organs such as kidneys, brain, reproductive organs, and liver (5, 6). Common sources of lead poisoning are in the car battery industry, manufacturing of ceramic, plumbing, primary and secondary smelting, and exposure to lead-bearing paint or contaminated food, water, and fuel (6, 7). The battery industry is one of the major sector sources of lead exposure (8). Seventy percent (70%) of the world's lead (Pb) production existed in the battery manufacturing and recycling industries (9). Occupational exposure to lead is common in developing countries where most employers and employees are not aware of the adverse health effects (10). Studies found in Bangladesh showed that workers in lead-acid battery industries had a high level of blood lead and they are suffering from many illnesses attributable to lead toxicity (11). The study conducted in Iran revealed that neuropsychiatric and skeletal cases were common manifestations of chronic occupational lead poisoning (6). Long exposure to a high level of lead may result in memory impairment, increased reaction time, and inability to perceive information (12). According to the American Conference of Governmental Industrial Hygiene Threshold Limit Value and Biological Exposure Indices standards, the exposure limit value for inhalation of lead for 8 TWA is 0.05 mg/m^3^ and for blood lead the recommended biological exposure index is 200 μg/L (13).

A limited study was conducted on the level of blood lead concentration among workers engaged in battery factories in low and middle-income countries. Therefore, this study will improve workplace health and safety conditions of workers in this sector and serve as baseline data for further studies in this segment of the working population. Therefore, this study aimed to identify the pooled mean blood lead exposure level among lead battery manufacturing workers in low and middle-income countries from existing literature.

## Methods

The finding of this systematic and meta-analysis was reported based on the Preferred Reporting Items for Systematic Review and Meta-Analysis statement guideline (14).

### Searching strategy and information sources

The search methods considered the following electronic bibliographic databases: google scholar, PubMed, and other gray literature. The principal search terms and phrases were Lead, LEAD, Pb, “Blood lead level, Intoxications, poisoning, toxicity, pollution, exposure, contamination, “battery factories,” “battery industries,” “battery company,” and “battery manufacturing”.

The search strategies were developed using different Boolean operators. To fit the advanced PubMed database, the following search strategy was applied on February 18, 2022, at 4:45 PM. ((((lead[Title/Abstract]) OR (LEAD[Title/Abstract])) OR (Pb[Title/Abstract])) OR (lead(MeSH[Title/Abstract]))) OR (“blood lead level”[Title/Abstract]) AND (((((Intoxications[Title/Abstract]) OR (pollution[Title/Abstract])) OR (contamination[Title/Abstract])) OR (exposure[Title/Abstract])) OR (toxicity[Title/Abstract])) OR (poisoning[Title/Abstract]) AND ((((“battery company”[Title/Abstract]) OR (“battery factories”[Title/Abstract])) OR (“battery industries”[Title/Abstract])) OR (“battery industry”[Title/Abstract])) OR (“battery manufacturing”[Title/Abstract]).

This title was registered on the Prospero database CRD42022322827.

### Research question

What is the level of blood lead concentration among battery factory workers in low and middle-income countries?

### Study selection

Initially, all articles were exported into Endnote version 9 software and checked for duplication. The duplicated articles were removed. Two independent authors, AT and TA, have reviewed the title and abstract. Three authors; AB, BME, and MB have scanned the abstracts and full documents. The disagreement was handled based on established article selection criteria.

### Inclusion and exclusion criteria

All studies conducted in lower and middle-income countries since the 2000 year of publication were included. Because many industries in low and middle-income countries have expanded in recent years and this is why we have made this research the starting point. We used the World Bank country classification to categorize the economic level of countries (15). Those studies reported at least the mean and standard deviation of blood lead level published and unpublished gray literature in the English language was considered at the searching stage. Moreover, where the studies include community, children, and non-humans or animals were excluded from the systematic review and meta-analysis.

### Screening and quality assessment

In the screening process, primary studies were reported without the outcome of the interest, and methodological problems were removed. However, all articles that don't access free without payment at screening time were accepted and assessed for final inclusion. Besides, studies with low quality as the pre-setted parameters were omitted. All included articles were critically appraised using the cross-sectional Joanna Briggs Institute (JBI) quality assessment tool (16). The Joanna Briggs Institute's critical appraisal tools for quantitative studies contain appraisal criteria that address both the validity and reliability of a study. Two independent authors AT and TA assessed the quality of the study, methodological fitness, and finding validity. Specifically, in the inclusion criteria, the study subjects and settings, study design, study country, the validity and reliability of the exposure measurement, outcome measurement, and the objective and appropriate statistical analysis were critically appraised. With the team's joint discussions, the uncertainties were fixed. Publication bias was checked by funnel plot (subjectively) and by the construction of Begg tests (objectively). The outcome of Begg tests with a *p* > 0.05 was reported which means no publication bias.

### Data extraction

Data were extracted using an excel sheet form prepared and used to extract data from the studies included for assessment of study quality. The outcome of interest (the mean and standard deviation of blood lead level) data extraction format consisted of the first author's name, the study year of publication, study country, study design, and sample size.

### Statistical analysis

The meta-analysis was done using Comprehensive Meta-Analysis V.3 statistical software. The pooled estimate of the main outcome (mean difference) and a 95% CI, were reported for the main outcome. Each eligible study's characteristics were briefly described using a summary Table. The summary table mainly described the characteristics of the studies included and the main findings. Forest plots were used to present the meta-analysis results graphically. To see publication bias, a funnel plot and Begg tests were used. The presence of statistical heterogeneity was checked using the Chi-square test (Cochran *Q* test) at a *p* ≤ 0.05. Heterogeneity between the studies in effect measures level by using *I*^2^ statistics, and we considered an *I*^2^ value >75% to be a significant heterogeneity indicator (17).

## Results

### Studies included

**Figure 1:**
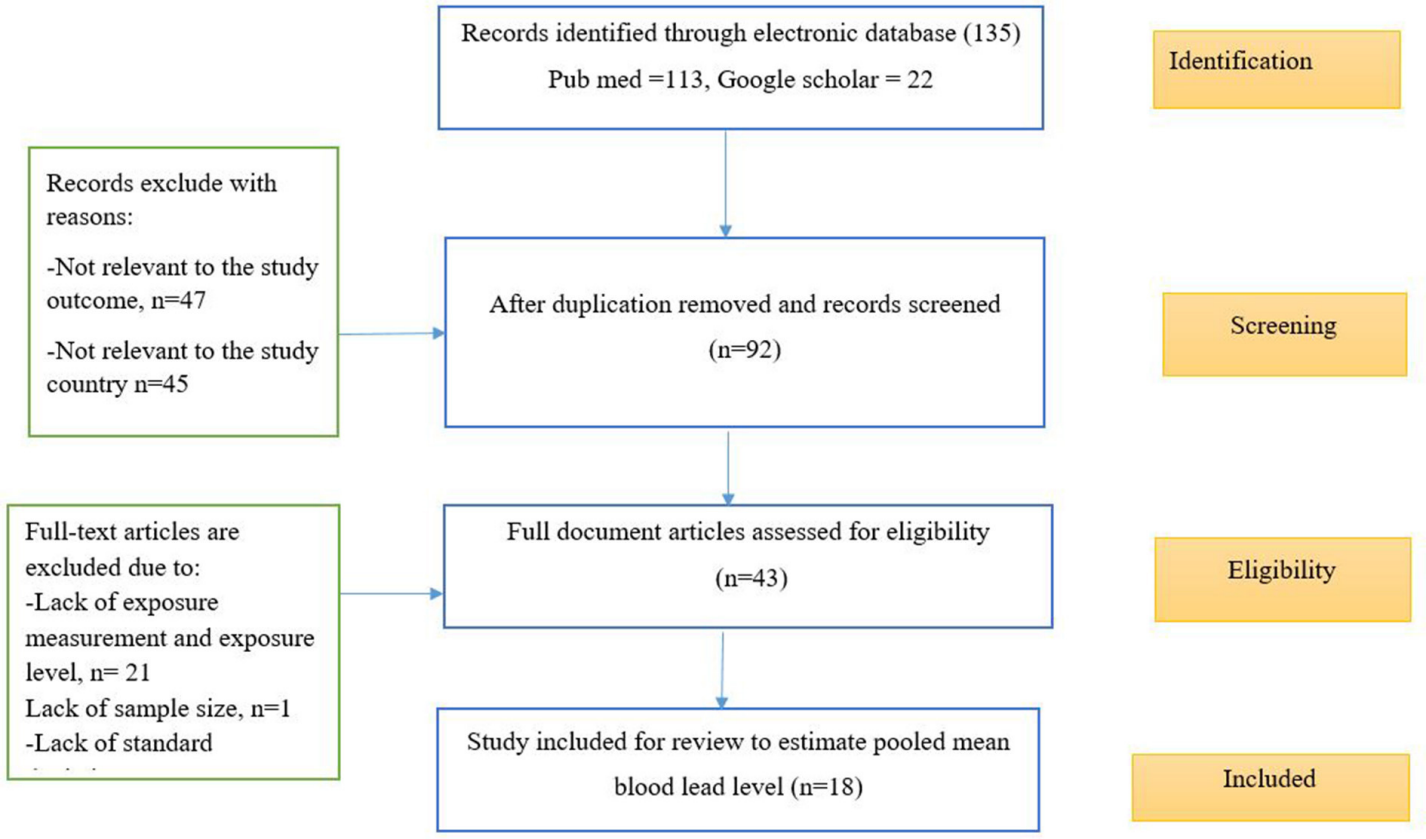
Article identification, screening, eligibility, and inclusion of studies on BLL among lead battery workers in LMIC, 2022 (*n* = 18).

An electronic database search: In the PubMed database search we identified 113 articles and in the google scholar search, 4,780 records were identified, of which the first (most relevant) 500 titles and abstracts were assessed, and finally 22 articles were identified and included in the screening criteria. Generally, 135 articles were found from a different electronic database, and all articles were imported into Endnote version 9.0 for duplication screening and full document review. Of 135 articles, 43 full-text articles were selected for a detailed evaluation of full document review. Finally, 18 studies remained after screening for inclusion-exclusion criteria and quality assessment. Figure 1 indicates the procedure of the article identification, screening, eligibility, and inclusion process.

### Study characteristics

The review included original studies with a total sample size of 2,736 lead-exposed works. All reviewed articles (18) were cross-sectional (10, 11, 18–33) study designs. Depending on the country distribution of the studies, nine studies were conducted in Iran (10, 18, 19, 21, 22, 25, 27, 28, 31), and six studies were from India (23, 24, 26, 30, 32, 33), 1 study from Egypt (20), 1 study from Tunisia (29), and 1 study from Bangladesh (11) lead battery manufacturing industries illustrated under Table 1.

**Table 1:** Description of the study characteristics included in the systematic review and meta-anlysis of blood lead levels among battery factories in LMIC, 2022 (*n* = 18).

**References**	**Country**	**Study design**	**Sample**	**Exposure**	**Exposure level**
			**Size (*n*)**	**Measurement method**	**Mean and standardevation [μ(*SD*)]**
Patil et al. (32)	India	Cross-sectional	28	Blood	Pb-B(μg/dL) = 53.63 ± 16.98
Patil et al. (33)	India	Cross-sectional	30	Blood	Pb-B(μg/dL) = 53.63 ± 16.98
Keramati et al. (18)	Iran	Cross-sectional	105	Blood	Pb-B(μg/dL) = 32.20 ± 13.70
Pourabdian et al. (31)	Iran	Cross-sectional	70	Blood	Pb-B(μg/dL) = 36.54 ± 4.34
Taheri et al. (19)	Iran	Cross-sectional	142	Blood	Pb-B(μg/dL) = 7.59 ± 2.75
Raafat et al. (20)	Egypt	Cross-sectional	42	Blood	Pb-B(μg/dL) = 52.40 ± 5.78
Aminian et al. (21)	Iran	Cross-sectional	113	Blood	Pb-B(mg/dL) = 41.41 ± 16.99.
Kianoush et al. (6)	Iran	Cross-sectional	112	Blood	Pb-B(μg/dL) = 39.89 ± 17.74
Ahmad et al. (11)	Bangladesh	Cross-sectional	118	Blood	Pb-B(μg/dL) = 65.25 ± 26.66
Kalahasthi et al. (23)	India	Cross-sectional	391	Blood	Pb-B(μg/dL) = 27.60 ± 11.40
Chinde et al. (24)	India	Cross-sectional	200	Blood	Pb-B(μg/dL) = 30.10 ± 4.13
Sadeghi et al. (25)	Iran	Cross-sectional	44	Blood	Pb-B(μg/dL) = 26.57 ± 5.24
Ghanwat et al. (26)	India	Cross-sectional	43	Blood	Pb-B(μg/dL) = 59.93 ± 9.57
Dadpour et al. (10)	Iran	Cross-sectional	138	Blood	Pb-B(μg/dL) = 39.89 ± 17.74
Ghiasvand et al. (28)	Iran	Cross-sectional	609	Blood	Pb-B(μg/dL) = 37.85 ± 17.55
Sadeghniiat-Haghighi et al. (17)	Iran	Cross-sectional	425	Blood	Pb-B(μg/dL) = 34.70 ± 16.70
Nouioui et al. (29)	Tunisia	Cross-sectional	52	Blood	Pb-B(μg/dL) = 7.53 ± 2.71
Kumar et al. (30)	India	Cross-sectional	100	Blood	Pb-B(μg/dL) = 39.50 ± 31.90

### Methods of exposure assessment

In all studies, exposure measurement methods were considered as primary inclusion criteria. Blood, urine, semen, air, bone mineral density, hair, and noise samples were taken from workers exposed to lead battery-manufacturing industries. All biological exposure index measurements (blood, urine, hair, semen, airborne, and bone) were extracted from the studies. However, except for the blood samples others were not used in all studies, therefore for this review only the blood lead levels exposure measurement were considered in all exposed workers (2,736).

### The pooled mean blood lead level of battery factory workers

**Figure 2:**
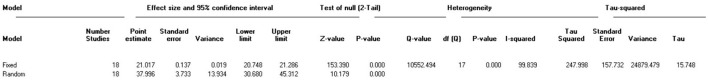
The summary of pooled mean BLL and the heterogeneity of the study among lead battery workers in LMIC, 2022 (*n* = 18).

The summary of the statistical pooled mean effect size and heterogeneity results is described in Figure 2. The overall pooled estimate of the mean blood lead level among battery factory workers in low and middle-income countries was 37.996 μg/dl (95% CI: 30.68–45.30) with a *p*-value of 0.000. The *I*^2^ value is 99.839, which showed that about nearly all of the variance in observed effects reflects differences in true effect sizes. The value of *I*^2^ is 99.839% which is >75% (17), therefore, this study suggests that there is heterogeneity. Therefore, we used the random effect model for this systematic review and meta-analysis study.

### Subgroup analysis

**Figure 3:**
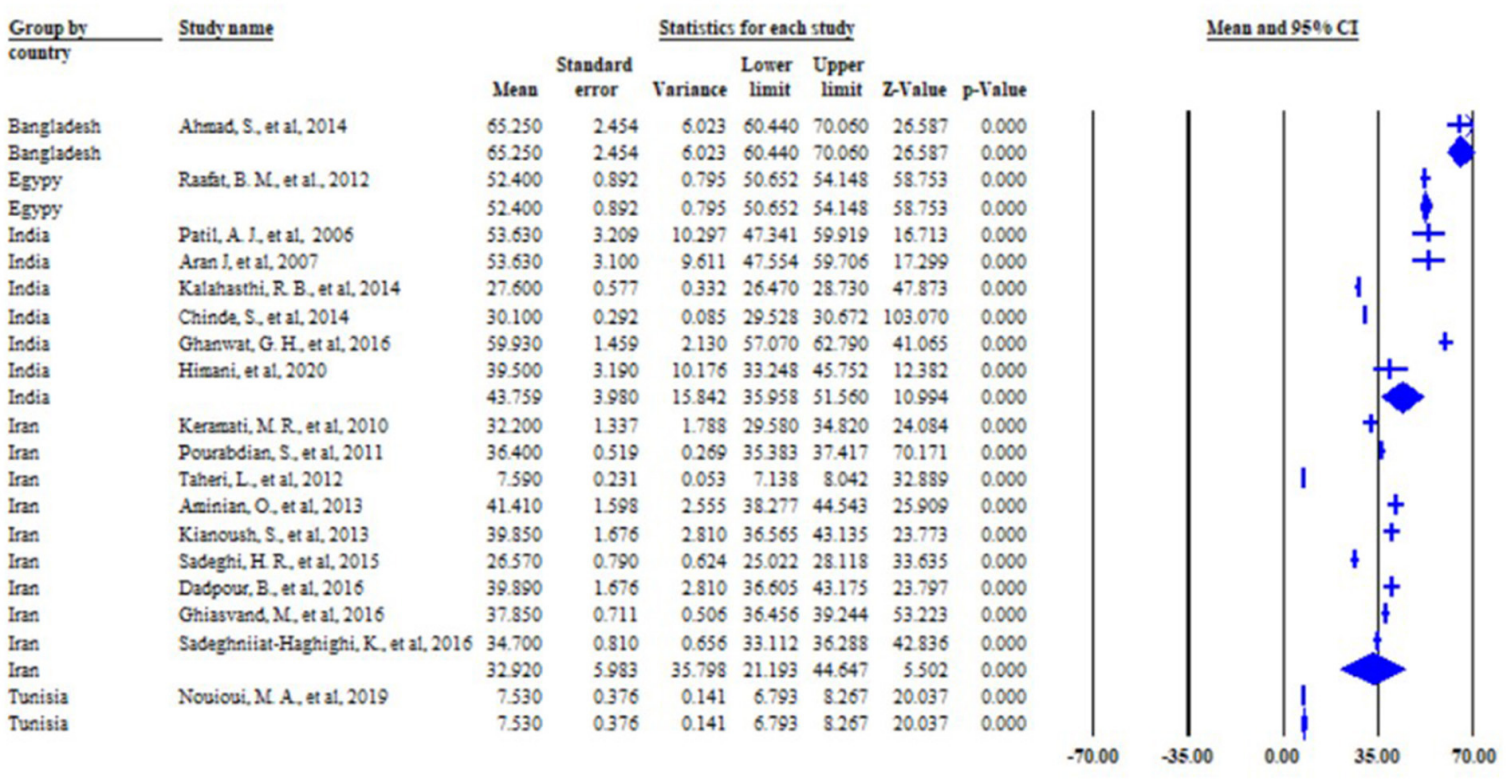
Figure subgroup analysis by the country level of the mean of BLL among lead battery workers in LMIC, 2022 (*n* = 18).

**Figure 4:**
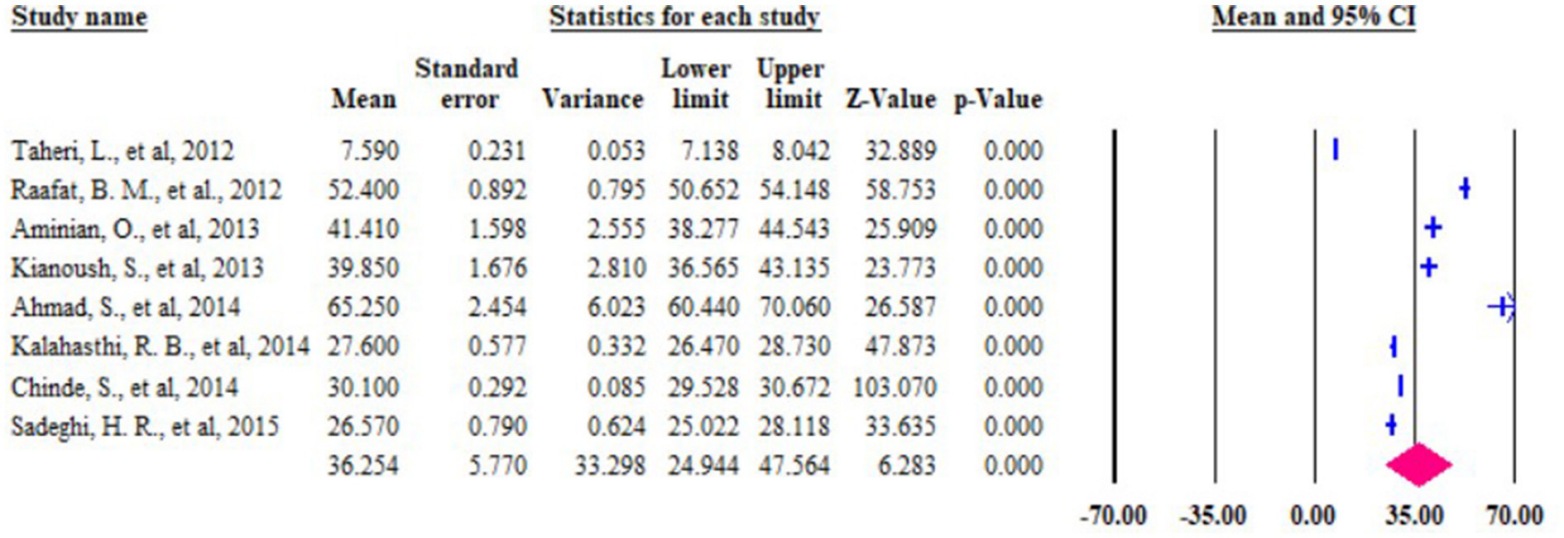
Figure subgroup analysis by year of the publication of the mean of BLL among lead battery workers in LMIC, 2022 (*n* = 18).

**Figure 5:**
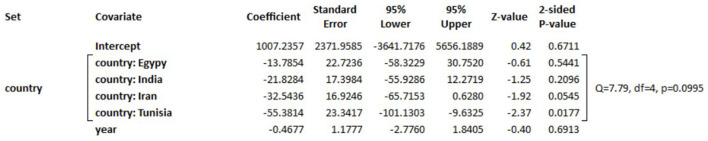
Meta-Regression of the pooled mean BLL among lead battery factory workers in LMIC 2022 (*n* = 18).

The review found that the *I*^2^ = 99.839%. This indicates high heterogeneity, so we performed a subgroup analysis by considering the study country and year of the study. Based on the subgroup analysis, the highest mean blood lead level was reported from Bangladesh (65.25 μg/dl) followed by Egypt (52.40 μg/dl). In addition, there was the highest BLL reported in the year 2006–2011 (43.21 μg/dl) followed by 2016–2020 (36.53 μg/dl) in the subgroup analysis report (Figures 3, 4 and Table 2) depicts the subgroup analysis by country level and in a year of publication. In addition to subgroup analysis, we performed Meta-regression analysis by including 18 studies to identify factories for heterogeneity. However, there was no statistical significance value from the meta-regression model (Figure 5).

**Table 2:** Pooled mean of blood lead level, 95% CI, and heterogeneity estimate with a *p*-value and *I*^2^ for the subgroup analysis.

**Variables**	**Category**	**A pooled point estimate of mean BLL(μg/dl) with 95% CI**	***I*^2^ (*P*)**
Country	Bangladesh	65.25 (60.44–70.00)	0.00 (1.00)
	Egypt	52.40 (50.65–54.15)	0.00 (1.00)
	India	43.76 (35.96–0.51.56)	99.06 (0.00)
	Iran	32.92 (21.19–44.64)	99.848 (0.00)
	Tunisia	7.53 (6.79–8.27)	0.00 (1.00)
Year of	2006–2011	43.21 (35.91–50.51)	95.67 (0.00)
publication	2012–2015	36.25 (24.90–47.56)	99.91 (0.00)
	2016–2020	36.53 (19.44–53.62)	99.83 (0.00)

**Figure 6:**
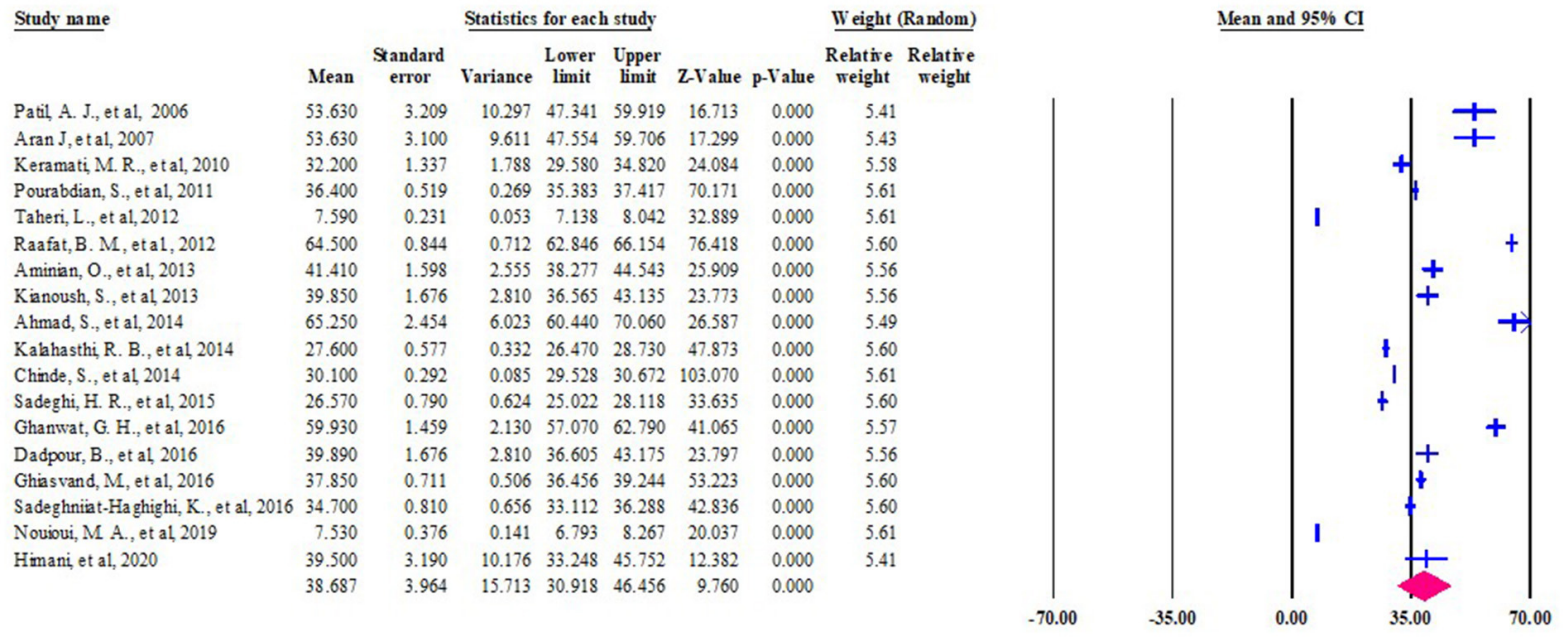
Forest plot of the pooled mean of BLL among lead battery workers in LMIC, 2022 (*n* = 18).

Figure 6 showed the forest plot and relative weight of the random effect model of the mean of blood lead intoxication among workers exposed to lead batteries in LMIC.

### Publication bias

**Figure 7:**
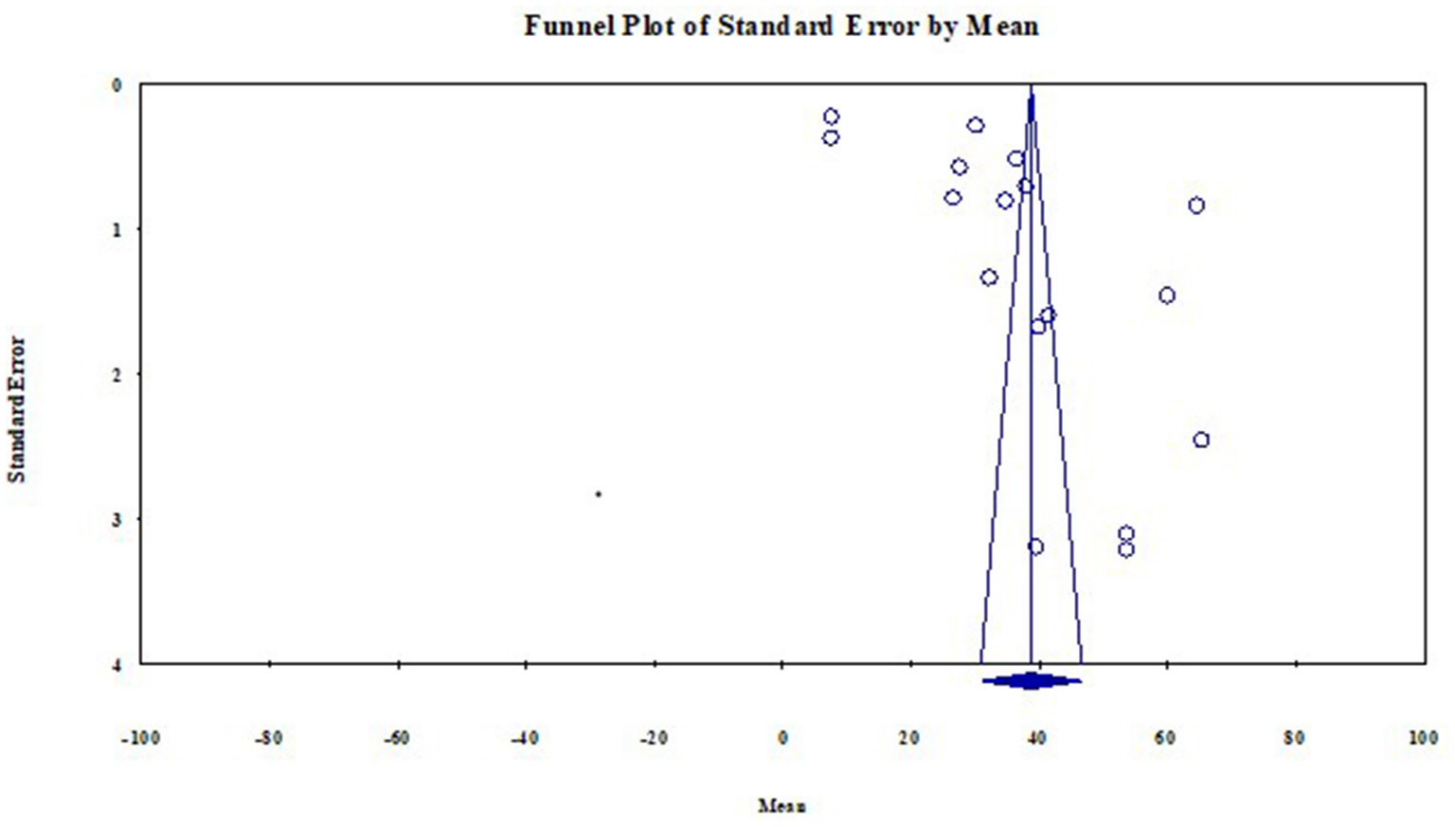
Funnel plot with 95% confidence limits of the mean BLL among lead battery factory workers in LMIC 2022 (*n* = 18).

This systematic review and meta-analysis identified that the studies' effect sizes are normally distributed around above the center of a funnel plot illustrated in Figure 7. Each study's scatter plot was clustered near pooled mean (37.996 μg/dl), suggesting no publication bias.

**Figure 8:**
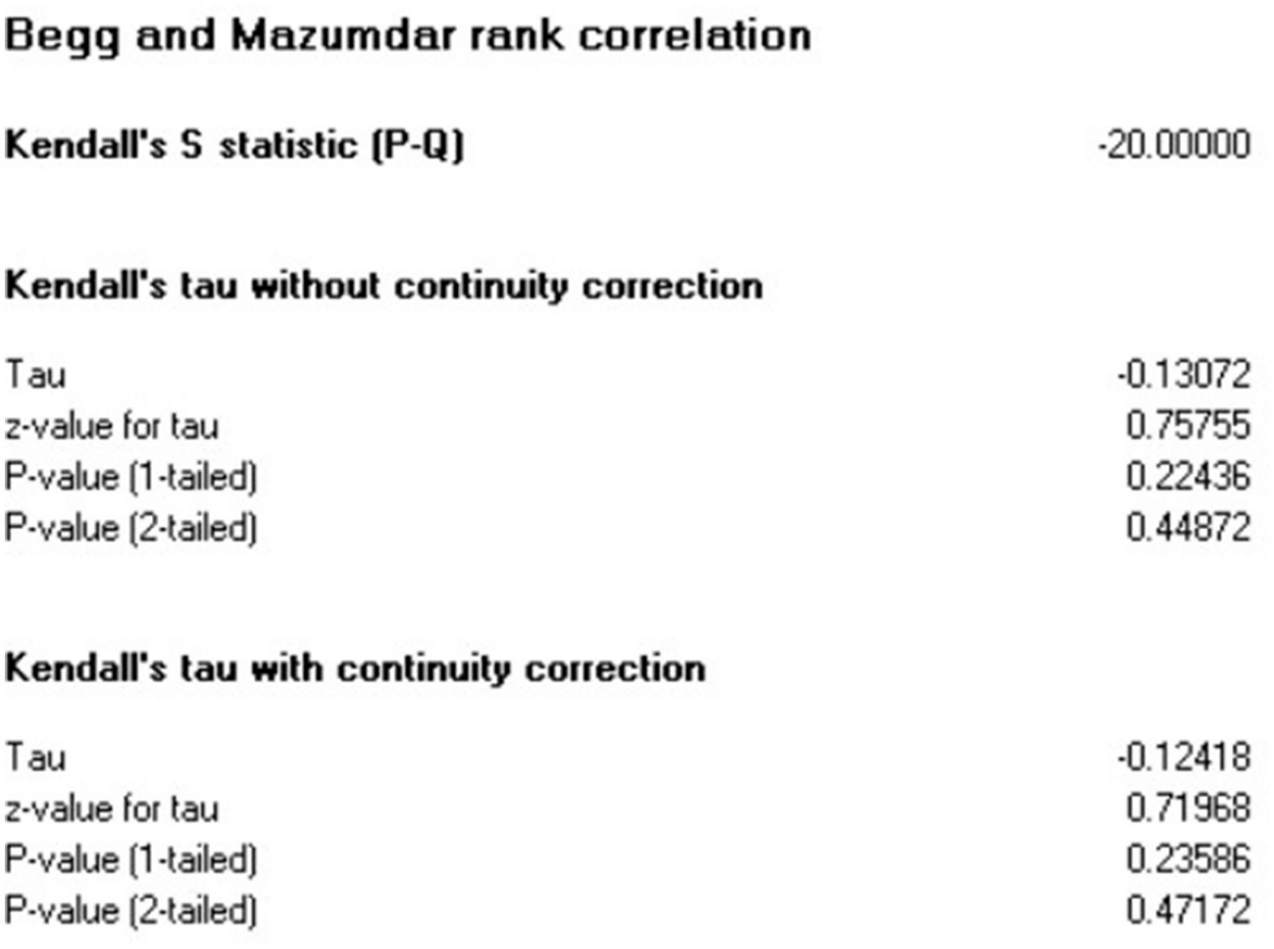
Egger's test and Begg and Mazumdar rank correlation publication bias test among lead battery factory workers studies in LMIC 2022 (*n* = 18).

The publication bias was objectively assessed using Begg's and Egger's tests to rule out no small-study effects. The estimated bias coefficient (intercept) using Egger's regression test was 24.60 with a standard error of 7.33 and a *p*-value of 0.0053. Egger's test provided evidence of the presence of publication bias with small study effects reported because the *p*-value is >0.05. However, in Begg and Mazumdar rank correlation test Kendall's tau continuity correction the *p*-value for 2-tailed is 0.47. This implies there is the absence of publication bias with small study effects. Therefore, for medium study size and continuous outcome variables, Begg's test is recommended. So for estimating the mean blood lead level among lead battery factory workers in Low and Middle-Income countries were [(*p* = 0.0053) and (*p* = 0.47) for Egger's test and Begg's test respectively] (Figure 8).

## Discussion

Every person in a productive age group spent one-third of his/her life at the workplace (34). Understanding the level of workplace exposure is a key issue to solving health problems raised in the workplace and from work activities, and to design appropriate prevention and control strategies, and improving the working conditions and working environments, which affect the health and safety of working populations. Scientific evidence is important to give directions to policymakers in the decision-making process. This systematic review and meta-analysis study identified the pooled mean blood lead level (37.996 μg/dl) among workers exposed to battery factory workers in low and middle-income countries. This finding is higher than the American conference of governmental industrial hygiene 2022 threshold limit value (TLV) which is 200 μg/L or 20 μg/dl (13). This shows that those workers engaged in battery factories in low and middle-income countries were exposed nearly two times above the international standard exposure limit value. Also, it is higher than a systematic review and meta-analysis conducted on the relationship between male reproductive and battery lead exposures[29.66 (95 % CI, 23.90–35.43)] (35) and nearly in line with a systematic review and meta-analysis conducted on the association of blood lead level exposure with markers of calcium homeostasis exposures [36.13 (95 % CI, 25.88–46.38)] (36). However, it is lower than the study conducted in Italy by lead battery storage workers [42.33 μg/dl] (37). The reason why this study was higher than this may be that it was done in only one area.

Based on the subgroup analysis, the highest pooled mean blood lead level was reported from Bangladesh (65.25 μg/dl) followed by Egypt (52.40 μg/dl). It is higher than the study conducted in Italy and South Korea [42.30 μg/dl, 32.00 μg/dl], respectively (37, 38). This high blood lead level reported might indicate that the occupational health service, the workplace exposure prevention, and control strategy designed by the country, the provision of appropriate personal protective equipment, and its utilization by workers may be low than in developed countries.

In the year subgroup analysis there is a high BLL reported in the year 2006–2011 (43.21 μg/dl) which is higher than the study conducted in among workers engaged in Turkeye lead battery factory workers (36.83 μg/dl) (39) and the result between the year 2016–2020 is 36.53 μg/dl, it is lower than study conducted in Pakistan 60.45 μg/dl (40). This indicates that the awareness of workers and employers on the prevention and control of workplace exposure somewhat improved compared to the current year with previous ones. However, still, the blood lead exposure level is above the international standard limit value. This showed that the provision of occupational health and safety services, labor inspection services, and the implementation of national and international legislation and standards is very low. Therefore, the government, international labor organizations, employers, workers' trade unions or representatives, and other stakeholders should be given attention to workplace health and safety to improve the health of the working population in low and middle-income countries.

## Strengths and limitations

The strength of the review was:

- The outcome of interest (the mean blood lead level) was measured by using biological exposure indexes (BEI) other than self-reported data.

The limitation of this review was:

- It is only focused on articles published in the English language
- Only assessed limited electronic databases
- Only focuses on one study arm (not a comparative study).

## Conclusion

This systematic review and meta-analysis showed that the pooled mean blood lead level of workers exposed to lead battery factories in low and middle-income countries was nearly two times above the international threshold limit value. Therefore, attention should be given by policymakers to improving the provision of occupational health and safety services at the workplace. Employers also should be working to apply appropriate control strategies to improve the health and safety of their workers. Researchers should work on workplace exposure-related problems to provide further findings for prevention and control mechanisms. Generally, workplace health and safety improvement is needed among workers exposed to lead battery factories in low and middle-income countries by providing appropriate occupational health and safety services in this sector.

## Data availability statement

The raw data supporting the conclusions of this article will be made available by the authors, without undue reservation.

## Author contributions

AT, TA, and AK: participated in a developing the study design and protocol, literature review, selection of studies, quality assessment, data extraction, statistical analysis, interpretation of the data, and developing the initial drafts of the manuscript. All authors contributed to the article and approved the submitted version.

## Conflict of interest

The authors declare that the research was conducted in the absence of any commercial or financial relationships that could be construed as a potential conflict of interest.

## Publisher's note

All claims expressed in this article are solely those of the authors and do not necessarily represent those of their affiliated organizations, or those of the publisher, the editors and the reviewers. Any product that may be evaluated in this article, or claim that may be made by its manufacturer, is not guaranteed or endorsed by the publisher.

## Abbreviations

ACGIH: American Conference of Governmental Hygiene
BLC: Blood Lead Concertation
BEI: Biological Exposure Index
BLL: Blood Lead Level
JBI: Joanna Briggs Institute
LMIC: Low and Middle-Income Countries
PbB: Lead concentrations in Blood
TLV: Threshold Limit Value
TWA: Time Weighted Average.
